# Which Combination Therapy for Uncomplicated Malaria in Africa?

**DOI:** 10.1371/journal.pmed.0020236

**Published:** 2005-07-26

There are at least 300 million acute cases of malaria each year globally, resulting in more than a million deaths. Ninety percent of deaths due to malaria occur in Africa, south of the Sahara, mostly in young children. The number of deaths is increasing, and one key factor linked to this has been widespread drug resistance of *Plasmodium falciparum* to conventional antimalarials, such as sulfadoxine-pyrimethamine (SP); such resistance is widespread in southeast Asia, South America, and Africa. The inappropriate use of antimalarials during the past century has contributed to this increase in resistance. For example, there has been overreliance on quinolines (such as chloroquine) and antifolates (such as pyrimethamine) resulting in cross-resistance among these drug classes. However, in the past decade, a new group of antimalarials—the artemisinin compounds, such as artesunate, artemether, and dihydroartemisinin—have been deployed on an increasingly large scale.

These compounds produce a very rapid therapeutic response, are active against parasites resistant to multiple drugs, are well tolerated, and reduce gametocyte carriage. To date, no parasite resistance to these compounds has been detected.[Fig pmed-0020236-g001]

**Figure pmed-0020236-g001:**
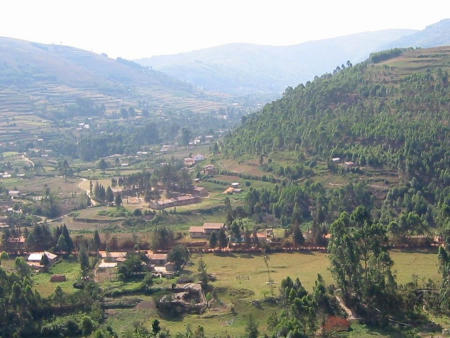
A trial site in Uganda

If used alone, the artemisinins will cure falciparum malaria in seven days, but studies in southeast Asia have shown that combinations of artemisinin compounds with certain synthetic drugs produce high cure rates after just three days of treatment. There is also some evidence that combinations of therapies could greatly retard development of resistance to the partner drug. Although combinations including artemisinins have been widely advocated, they are expensive and relatively untested in highly endemic areas.

In this month's *PLoS Medicine*, Adoke Yeka and colleagues compared artemisinin-based compounds and other combination therapies in four districts with varying transmission intensity in Uganda in 2,160 patients aged six months or greater with uncomplicated falciparum malaria. The team tested the combination of chloroquine and SP, currently the first-line therapy in Uganda, the combination of amodiaquine and SP, a cheap regimen proven to be efficacious in previous trials, and the combination of amodiaquine and artesunate.

During the 28-day study they collected data on the efficacy of the different regimens and examined the effect on recrudescence and new infections after therapy. Combined amodiaquine and artesunate was the most efficacious regimen for preventing recrudescence, but this benefit was outweighed by an increased risk of new infection. This result was probably due to artesunate being rapidly eliminated, leaving only amodiaquine to provide post-treatment prophylaxis. Considering all recurrent infections, the combination of amodiaquine and SP was at least as efficacious as the other combinations at all sites and superior at the highest transmission sites.

In all, 72% of all recurrent infections were due to new infections, and with the two most efficacious regimens (amodiaquine and SP, and amodiaquine and artesunate) this proportion was 80%. The identification of new infections stressed the need for other malaria control measures, such as bed nets, said the authors.

They also suggested that antimalarials should be judged not just on their impact on recrudescence but also on their impact on the risk of new infections after therapy. Previous studies have suggested that patients who suffer recrudescence have a higher risk of complicated malaria and death. Artemisinins are highly attractive antimalarials, but when used as monotherapy, they have a high risk of recrudescence and hence must be combined with other antimalarials to achieve maximum efficacy. But whether the partner drug should be long or short acting remains unclear, said the authors.

Altogether, artemisinin combinations offer great hope for Africa, the authors say, although the ideal combination regimen remains uncertain and cost is a problem. To compare the efficacy of the different therapies, bigger and longer controlled trials are needed in conditions of varied transmission intensity. Nevertheless, based on the results of this study and others, Uganda has chosen a combination of artemether and lumefantrine as its first-line therapy against malaria.

